# The Treatment of External Cancer with Caustic Potash

**Published:** 1901-05

**Authors:** M. B. Hutchins

**Affiliations:** Atlanta, Ga.


### THE TREATMENT OF EXTERNAL CANCER WITH CAUSTIC POTASH.*

BY M. B. HUTCHINS, M.D.,

ATLANTA, GA.

In other papers I have referred to the use of this chemical in the treatment of skin cancer. Quite a large further experience with it

* Read before the Medical Association of Georgia, April, 1901.

has but confirmed my opinion of its value. My reason for using the words "external cancer" in the title is that I have employed the caustic potash in other than simple epitheliomatous cases. I do not believe there is a "cure-all" for cancer and have never been willing to accept any one method of treatment as the only one.

The treatment of cancer with success depends simply upon our ability to remove or destroy all the disease, as I have always maintained. The more conveniently and agreeably this can be done and the better looking scar we can get, the more satisfactory is our treatment to the patient.

Caustic potash is the "hydrate or hydroxide of potash" and is a powerful alkali. The molded stick deliquesces from the absorption of moisture when exposed to the air. Vegetable acids constitute its surest antagonist. The National Dispensatory, from which I get a description of the chemical, says "it destroys animal tissue by abstracting water, neutralizing free acids, decomposing nitrogenous compounds and forming solutions of fibrin, albumen and gelatin."

It acts more rapidly upon epithelial structures than on the more vital tissues, and hence more destructively upon the cells of cancer than upon normal structures. The addition of moisture is essential to its action upon very dry epithelial growths. It cuts the blood-vessels and is able to stop the resultant bleeding only through the mechanical plugging afforded by the gelatinous, destroyed mass. It does not contract the vessels nor coagulate fibrin, if any fail to yield to its destructive action. In my efforts immediately to remove the soft mass of destroyed tissue from a lady's face I brought on bleeding, which persisted, with intervals, for two hours. The blood was of low coagulability.

When it becomes necessary to neutralize the caustic potash I use acetic acid. It is also used around the diseased area to protect healthy structures. In using the caustic in the corner of the eye, even between the eyeball and inner wall of the orbit, I have always been able to prevent injury to the eye by the timely use of acetic acid.

The use of arsenic to destroy cancerous growths is very old. It is corrosive and has been credited with special selective action on the disease. Its usual method of application by reputable practitioners is in the form of Marsden's paste, the average formula of which is equal parts of arsenious acid and powdered gum acacia made into a paste with water. It must remain in contact with the disease for from ten to twenty-four hours, and is painful all during that time. On a large surface there is danger of absorption, and it is also unsafe near the eye or mouth. Having these unpleasant features, and not having given me any better, if as good, results as caustic potash, I have given it up.

Chloride of zinc is very painful, a coagulant and makes a tough, dry eschar. It acts like the acid caustics and has no affinity for epithelial growths. I have never used it.

Acid caustics coagulate the tissues and the blood, constricting the vessels. The pain is intense with some of them, the eschar tough and the scar likely to be contracted, hard and ridged. Sulphuric acts like the cautery. The actual or electro-cautery I no longer find indications to use. The glowing metal does not reassure the patient and the effect does not justify its use, so long as caustic potash can be had.

Other caustics need not be mentioned, as they have no special claim to be used.

When applied to the average brittle epithelioma, in which there is not much fibrous tissue, or to a lesion which is succulent, caustic potash rapidly changes it to a black, jelly-like substance. The fibrous tissue in and around the lesion is necrosed, becoming grayish but not so soft as the less stable substances. The destruction is almost instantaneous, if sufficient moisture be present.

The pain is severe but begins to subside in one or two minutes, as a rule, being entirely gone by the end of five minutes. Of course there are exceptional cases in which the time varies. Once having ceased the pain never recurs, while nitric acid, or the acid nitrate of mercury, for example, sometimes produce persistent or paroxysmal pain which may last several hours. Even nitrate of silver may cause a stinging sensation lasting half an hour or more.

The injection in and around the tissues of a one to two per cent. solution of cocain, or the application during several minutes of a 10 per cent. solution to a raw surface, unfortunately exercise but little anesthetic effect against caustic potash. A saturated solution of eucaine *B* does somewhat better. Most of my cases having been of the face or head, I have been afraid to use much cocain, not caring to have a death under any anesthetic or operation. In cases where the application of the caustic has to be prolonged or extensive I employ general anesthesia.

If the lesion to be treated is small and any part of the surface is broken, I usually employ the deliquesced caustic potash on a tooth-pick or probe, having cotton twisted on the end, boring in as deeply as necessary. The "deliquesced" is obtained by putting the stick caustic in a bottle, stopping it up only with cotton which permits the entrance of sufficient moisture from the air gradually to liquefy the stick to the consistence of mucilage.

The deliquesced form is used in most cases because it is more easily kept in bounds. If the stick is used one must watch carefully to prevent overflow from its rapid liquefaction which might cause serious damage to adjacent structures.

If I have an unbroken surface lesion of some extent, I usually break in or curette away the redundant tissue before using the caustic. If the lesion is large and in a region where the action of the caustic must be very deep and extensive, I use the stick caustic potash, wrapping all but the end to be used in tinfoil to protect the fingers. The stick is thoroughly bored in, rapidly dissolving as it unites with the substances attacked. The surrounding tissues are protected with acetic acid.

Where I have to treat a subcutaneous cancerous nodule, usually metastatic, I first, under cocain, split the skin over the nodule then inject eucaine *B* solution and bore in with the caustic.

It is best to leave the necrosed mass *in situ*, as nothing is gained by its immediate removal and troublesome bleeding may follow. Generally a dry powder, as subnitrate of bismuth, is put on, which helps the appearance a little and aids the air in drying the mass. The dried eschar prevents unpleasant oozing and overflow from the treated surface. Plain, or salicylated, rubber adhesive plaster or the constant presence of a grease or simple salve help to hasten the loosening of the eschar, and I often finish the separation with a pair of scissors, without pain or bleeding.

Any small remainder of eschar soon comes away and the wound heals by granulation, under treatment by proper methods, leaving a small and not unpleasing scar. The smooth, thin scar left after the use of caustic potash is one of the valuable points in its favor. Its special action on epithelial structures accounts for its almost selective effect upon the disease in epithelioma.

Caustic potash is applicable in any circumscribed and non-metastatic cancer where it is impracticable to use the knife, and there are many situations where it is not well to use the knife because of the danger of cutting into the growth and transplanting the disease to healthy tissues. I consider the knife as efficacious where there is plenty of room for wide excision. Whether caustic potash produces an inflammatory wall which chokes outlying cells I am not prepared to say.

In some cases I have found it best first to destroy diseased tissues with caustic potash, then, after a healthy surface appears, do the plastic operation required for repair of the defect.

In many cases caustic potash is preferable to the knife because it simplifies the preparation for treatment, does not run counter to the patient's prejudices, and because it destroys absolutely all diseased tissue which it meets. The after-treatment is however much longer than after an excision, provided excision removes all the disease. The ideal treatment of localized skin cancer would be, in all cases, to destroy the disease with caustic potash and as soon as inflammation subsides and a clean wound appears, do an excision followed by the proper fitting and stitching to make a good cosmetic result and hasten the end of treatment.

Caustic potash is preferable to arsenic paste because of its far quicker action and the possibility of keeping the patient under observation during the time it is employed and because it is safer and as efficacious.

The description of the action of some of the other caustics, as given above, shows sufficiently the superiority of caustic potash.

But brief reference will be made to cases. I have a great number of small cases on record which were treated with caustic potash, remaining well to this time after treatment given from one to seven years ago. A remarkable case was that of an old gentleman whom I did not wish to treat. Under my direction he went to an expert with the arsenic paste, in another city, and was pronounced cured in three or four weeks. The disease had occupied the left side of the nose and the cheek adjacent and part of the upper lip. A year and a half after the arsenic treatment he returned to me with involvement of the cheek from near its middle third, the left side of the nose, the nostril and base of the upper lip on the left side. There was a separate lesion on the bridge of the nose and one on the right ala.

The disease was persistently treated, point by point, as the need arose, many lesions were cured, and the rest put in such condition that a little caustic potash now and then to a suspicious point kept it in control. There is good scar tissue and the patient in robust health—according to my last record of the case. The best of the treatment was given this patient nearly two years ago. He is now about 74 years old.

In November, 1899, I operated on an old lady for recurrent carcinoma of the chest wall, and for disease left by a previous operator in the subclavian and axillary regions. Everything was removed from the upper chest but the ribs and intercostal muscles. Carcinomatous nodules had to be dissected from the axillary vein and the whole axillary space was cleaned out. The axilla and the region of the neck and clavicle have shown no evidence of recurrent disease, but several pea- to finger-tip sized subcutaneous nodules have appeared from time time on the chest. These have been destroyed with caustic potash, the skin over them first being split. In this way I have been able to prevent the growth of those intractable nodules which often become large, diffuse and form extensive metastases.

A case of cancer of the lower and inner sides of the orbit and the cheek and nose adjacent has been kept under control with caustic potash and seems in a fair way to get well. The treatment has been conducted without the slightest injury to the eye.

These cases with unfavorable prognoses have been reported as an example of what can be done with bad cases by proper treatment and attention. There are many others on my records which serve to confirm my claims as to the utility of caustic potash, but it is not necessary to detail them here.

In conclusion, it may be well to warn against the too conservative treatment of any form of cancer, whether this conservatism shows itself in a fear to do anything, sympathy for the patient or dread of disfigurement. Leaving the disease alone is as dangerous as wrong treatment. The disfigurement of treatment is better than that of extensive disease, and the pain of the patient is better taken all at once rather than in broken doses for months or years, with nothing but a horrible death at the end.

No. 64 Marietta street.

